# Supplementary material for: Assessment on Different Vaccine Formulation Parameters in the Protection against Heterologous Challenge with FMDV in Cattle
Source: Viruses. 2022 Aug 15;14(8):1781. doi: 10.3390/v14081781 (PMC9416185; doi:10.3390/v14081781)
Supplement: Supplementary file 1 [file viruses-14-01781-s001.zip › viruses-1829449-supplementary.pdf]

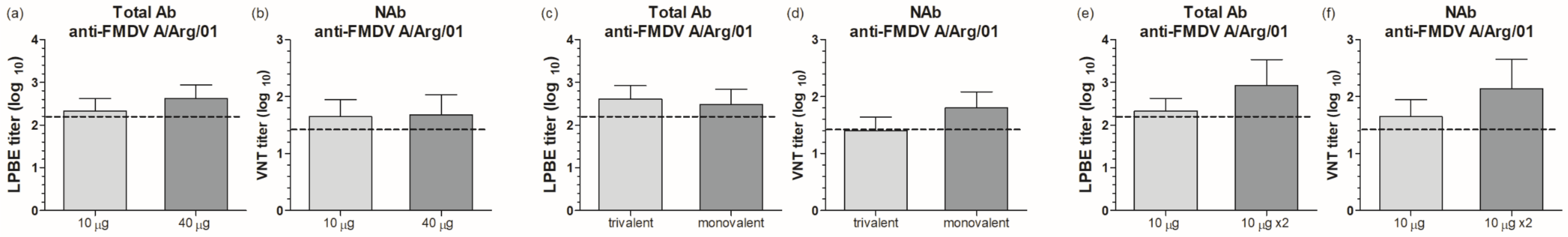

Figure S1. Total and neutralizing antibody titers against FMDV A/Arg/01 at 30 dpv according to the antigenic payload, strain composition or number of doses. Total FMDV-specific antibodies were measure by LPBE and neutralizing antibodies (NAb) were measure by VNT. Animals were grouped according to: (a and b) the antigenic payload of the formulation (10 µg/dose including the *A24 10µg group* vs. 40 µg/dose including the *A24/C3I/O1C* and *A24 40µg* groups), (c and d) the strain composition in the vaccine (trivalent including the *A24/C3I/O1C* group vs. monovalent including the *A24 10µg* and *A24 40µg* groups), and number of doses with the lowest antigenic dose (*A24 10µg group* vs. *A24 10µg x2* group). Results are expressed as the log_{10} of the mean titers against heterologous strain, A/Arg/01. Dotted lines depict the titers corresponding to the EPP_{75} for FMDV A/Arg/01 strain for each serological assay. Bars represent mean value ± SD.

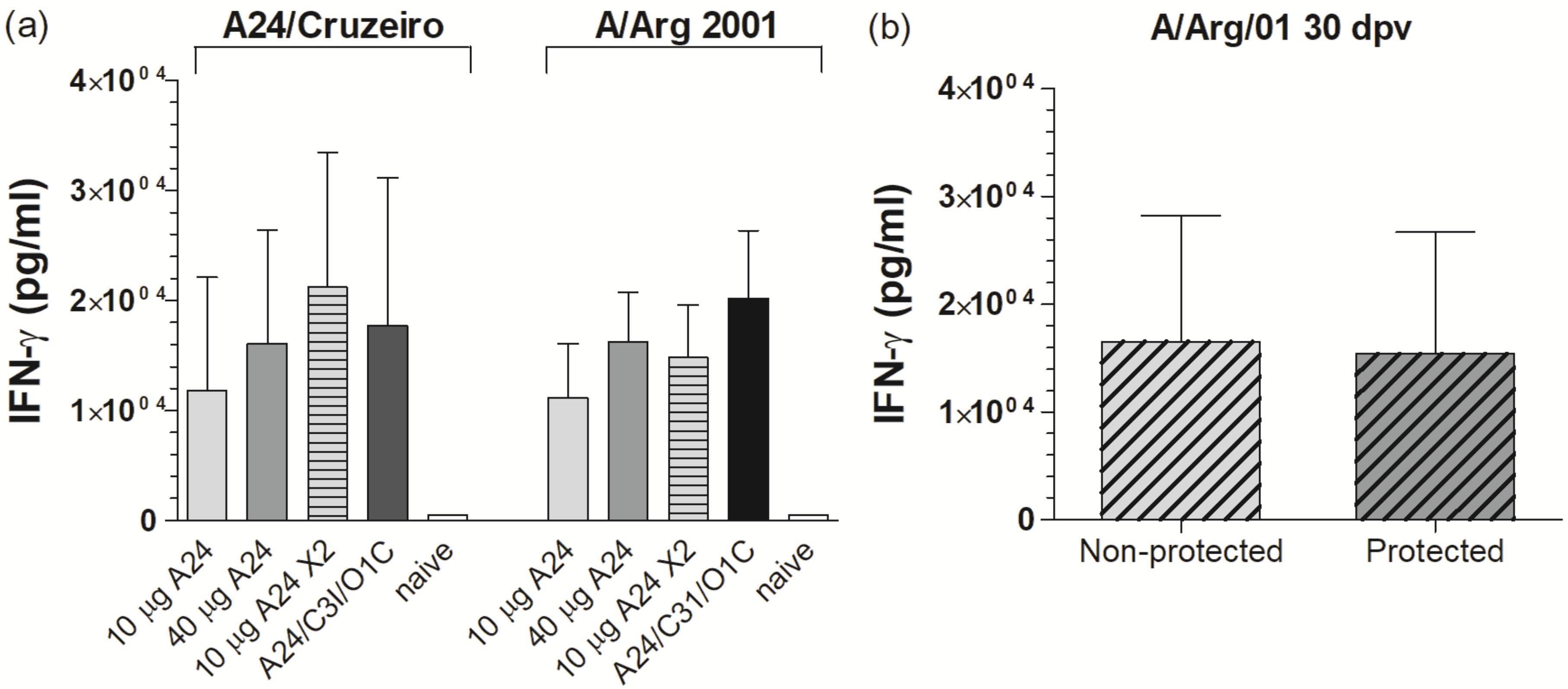

Figure S2. FMDV-specific interferon (IFN)-γ production per experimental group and clinical status after FMDV A/Arg/01 challenge. (a) *Ex-vivo* production of IFN-γ in experimental and control groups was measured by ELISA on stimulated plasma from whole blood samples incubated with 10 µg/mL of inactivated FMDV A24/Cruzeiro or A/Arg/0 strains, as indicated. (b) IFN-γ production in samples stimulated with the challenge strain, grouped according to the protective status after heterologous infection. Cell viability was assessed for each sample by incubating with 10 µg/mL of PWM (not shown). Bars represent the mean production of INF-γ ± SD for each group.

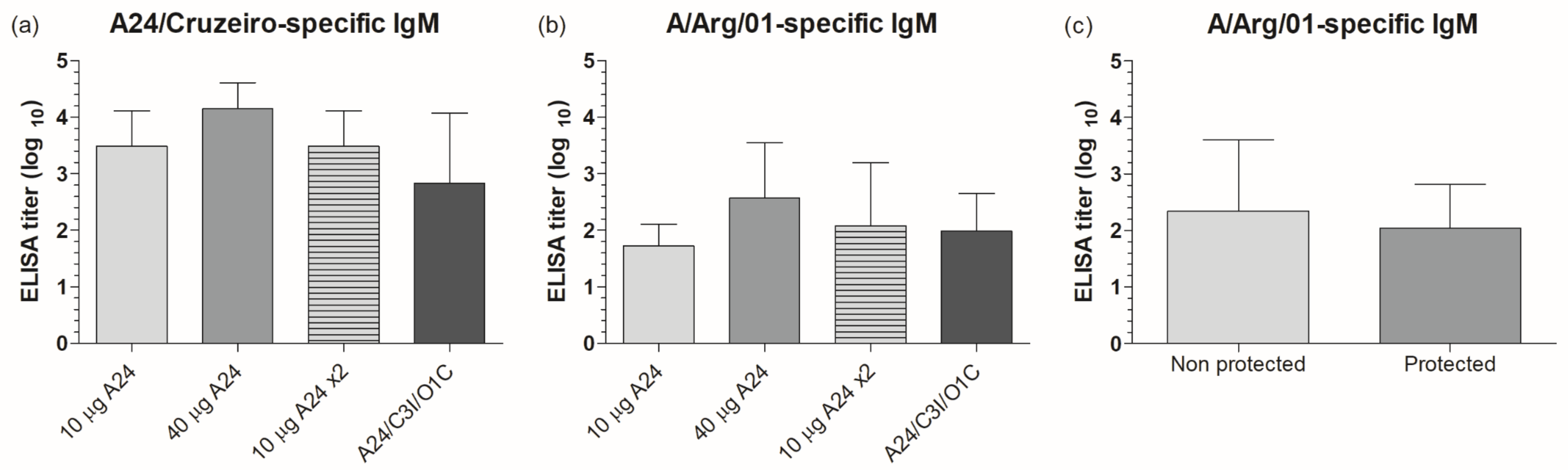

Figure S3. IgM titers against FMDV A24/Cruzeiro and A/Arg/01 strains per experimental group and clinical status after FMDV A/Arg/01 challenge. FMDV-specific IgM responses were measured by ELISA against the A24/Cruzeiro (a) and A/Arg/01 (b) strains. (c) Mean IgM antibody titers against the A/Arg/01 strain were also compared in animals grouped according to their protection status. Results are expressed as the log_{10} of the mean titer for each experimental group at 30 dpv, and bars represent the mean value of each experimental group ± SD.

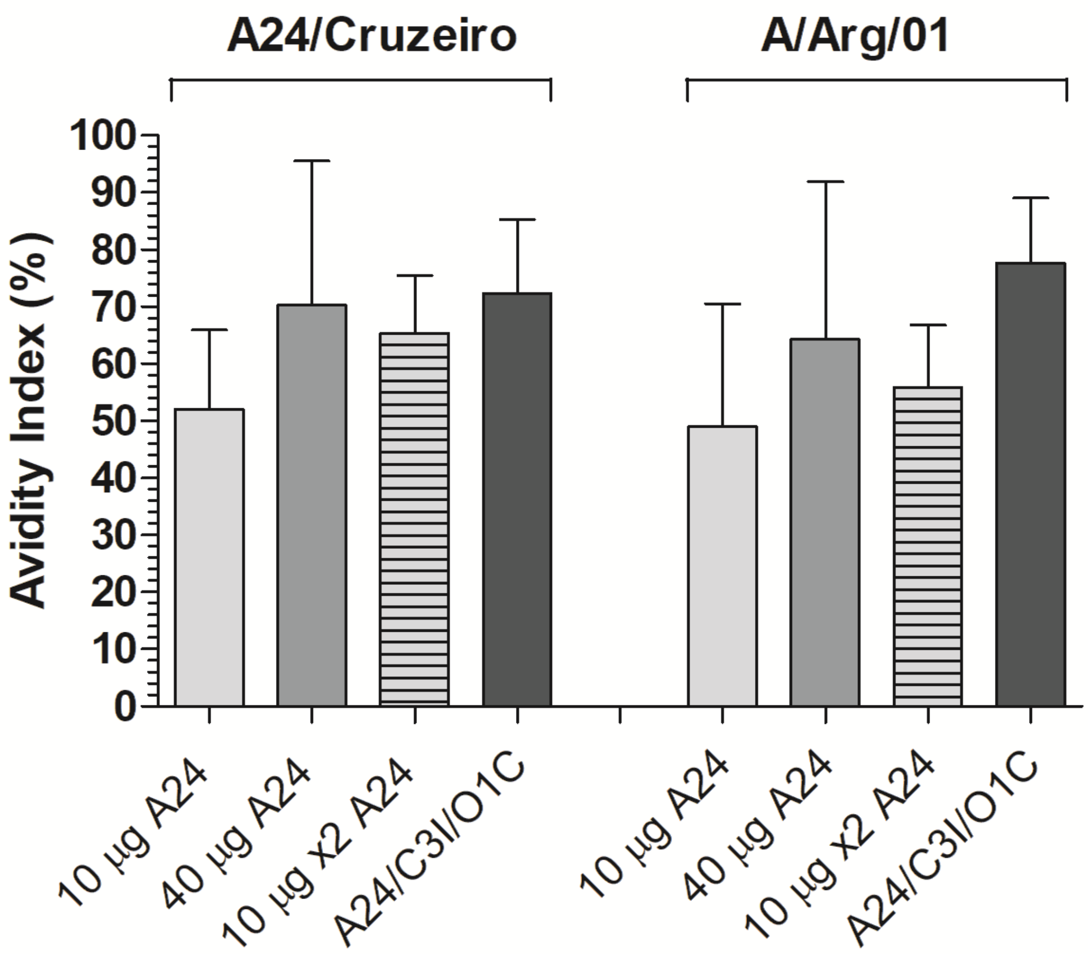

Figure S4. Avidity of the IgG antibodies against the FMDV A24/Cruzeiro and A/Arg/01 strains at 30 dpv per experimental group. The avidity of the FMDV-specific IgG antibody responses against the A24/Cruzeiro and A/Arg/01 strains were measured by Avidity ELISA. Results are expressed as the mean AI per experimental group and bars represent the mean value ± SD. Statistical differences between experimental groups were studied by ANOVA.

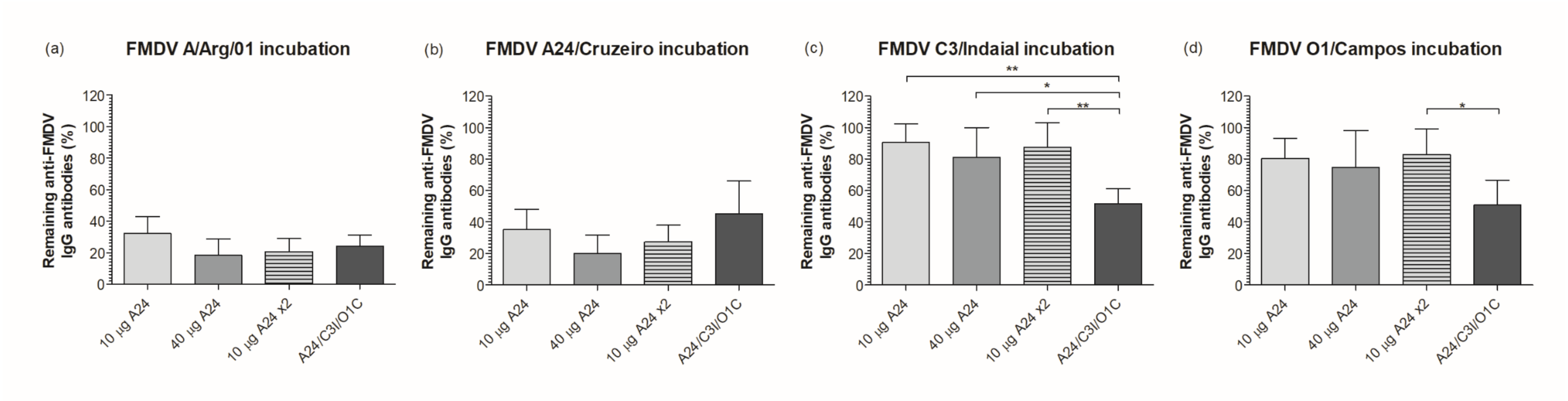

Figure S5. Percentage of total IgG antibodies against FMDV A/Arg/01 remaining in sera from vaccinated animals after incubation with different FMDV strains. Serum samples from vaccinated steers were incubated with PBS, or with inactivated purified FMDV antigens from the A/Arg/01 (a), A24/Cruzeiro (b), C3/Indaial (c) or O1/Campos (d) strains. Virus-incubated serum samples and their PBS-incubated replicates, were then evaluated for the presence of IgG antibodies against the A/Arg/01 strain by an indirect ELISA, using plates coated with purified inactivated FMDV A/Arg/01 (see Appendix A for methodology details). Results are expressed as the percentage of residual activity of the serum sample after virus incubation, relative to that of PBS-incubated sample (OD sample incubated with virus/OD sample incubated with PBS × 100). Bars represent mean values ± SD per experimental group. Asterisks denote statistical differences between experimental groups (ANOVA) * $p \leq 0.05$, ** $p \leq 0.01$.
